# *Enterobius vermicularis* in the Appendiceal Lumen: A Case Report

**DOI:** 10.1155/2024/7806541

**Published:** 2024-10-15

**Authors:** Mehta Razzaghi, Hosna Rezaei, Farshid Mohammadi, Amir Mohammad Salehi

**Affiliations:** ^1^Clinical Research Development Unit of Besat Hospital, Hamadan University of Medical Sciences, Hamadan, Iran; ^2^Internal Medicine, Clinical Research Development Unit of Shahid Beheshti Hospital, Hamadan University of Medical Science, Hamadan, Iran; ^3^Student Research Committee, Hamadan University of Medical Science, Hamadan, Iran

**Keywords:** appendectomy, appendicitis, *Enterobius vermicularis*, pinworms

## Abstract

*Enterobius vermicularis* is a common parasitic infection worldwide. Acute appendicitis (AA) is a frequently encountered condition in general surgery; however, its association with *E. vermicularis* remains controversial. AA caused by *E. vermicularis* is a relatively uncommon infection that primarily affects children. We reported a 21-year-old female who was admitted to our hospital due to right lower quadrant abdominal tenderness. Laboratory tests and imaging were not consistent with AA. She underwent a diagnostic laparoscopy and appendectomy. Histopathological examinations revealed the presence of *E. vermicularis* in the lumen of the appendix, which caused its obstruction without evidence of inflammatory cell infiltration or lymphoid hyperplasia.

## 1. Introduction

The most frequent abdominal emergency worldwide is acute appendicitis (AA) [1]. AA is a serious medical condition that occurs when the appendix becomes inflamed due to a blockage inside it. This condition causes severe abdominal pain and requires immediate medical attention [2].

The common symptoms of AA include vague periumbilical pain that migrates to the right lower quadrant of the abdomen over time, loss of appetite, vomiting, and low-grade fever. The diagnosis of AA relies on the patient's medical history, physical examination, laboratory analysis, and abdominal imaging [3, 4]. A wide spectrum of cases has been implicated as causes of AA, such as fecal stasis, fecaliths, lymphoid hyperplasia, undigested vegetable residues, fruit seeds, colorectal cancers (specially cecal cancer), and intestinal parasites [5–7]. Infection of the appendix itself by parasites is rather rare. Some parasites, such as *Schistosoma* spp., *Taenia* spp., and *Ascaris lumbricoides*, are known to cause AA [5, 8]. However, medical professionals have been discussing whether parasitic infestation could be linked to AA. Most of the reported cases of AA have occurred in the pediatric population, with few cases reported in adults [1, 3]. Herein, we presented a case of an adolescent woman diagnosed with AA, in which *E. vermicularis* was found in her resected appendiceal specimen.

## 2. Case Presentation

Our patient was a 21-year-old female with no significant past medical or surgical history who presented to the emergency department at Besat Hospital in Hamadan, Iran, complaining of right lower quadrant abdominal pain of 5 days' duration. The pain was progressive with associated anorexia and fever. She denied any changes in her bowel habits and menstruation, with her last menstrual period occurring 2 weeks ago.

On examination, her vital signs were as follows: blood pressure: 120/80 mmHg, pulse rate: 80 b/min, body temperature: 38°C, and respiratory rate: 25 b/min. Her abdominal examination revealed tenderness in the right lower quadrant with a negative rebound sign; also, no organomegaly was detected. Laboratory examination showed a hemoglobin level of 11.8 g/dL, a white cell count of 4700, and C-reactive protein levels of <4.0 mg/L. Urinalysis indicated no infection, and the pregnancy test was negative. Additionally, the coagulation profile and renal function tests were normal.

The pelvic ultrasonography of the patient revealed normal ovaries without any free fluid. As the likelihood of AA was low, a computed tomography scan was conducted, which showed that the appendix was retrocecal with a wall thickening of 7 mm in a short segment. However, there were no notable indications of fat stranding, which suggested borderline tip appendicitis. Therefore, a diagnostic laparoscopy and laparoscopic appendectomy under general anesthesia were performed. Intraoperative findings revealed a mildly hyperemic appendicular tip that was adherent to the lateral abdominal wall and cecum, with minimal hemorrhagic fluid in the pelvis. Her postoperative period was unremarkable. Within 8 h, she passed flatus and started oral feeding.

**Figure 1:**
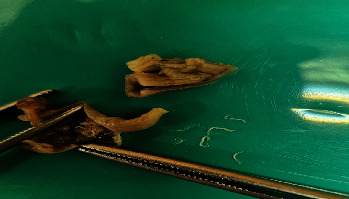
A gross image of the noninflamed appendix with *Enterobius vermicularis*.

**Figure 2:**
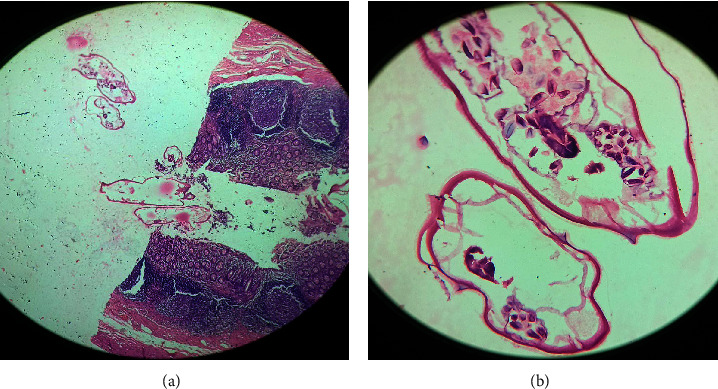
Histopathology showing luminal *E. vermicularis* with no evidence of an acute inflammatory process (H&E) (a); a gravid female parasite with colorless eggs and some organs (H&E) (b). H&E, hematoxylin and eosin.

In the microscopic view, the appendix appeared noninflamed, and a few worms were noted within the lumen (Figure 1). On histopathology examination, no evidence of lymphoplasmacytic or neutrophilic infiltration or mucosal ulceration was identified, and a normal appendix was observed. Worm larvae with a thick cuticle and visible eggs were identified in the lumen (Figure 2a). Cross-sections and several organs of the worms were visible. Colorless eggs, with one convex and one flattened side, can be seen inside the worms, revealing a thin, transparent hyaline shell (Figure 2b).

She was discharged home after 24 h with 400 mg of cefixime daily for 1 week and 400 mg of albendazole once weekly for 3 weeks (for the patient and her family). A follow-up visit was scheduled for 8 days later, at which time she was healthy, and the stitches were removed.

## 3. Discussion

Threadworm or pinworm, also known as *E. vermicularis*, continues to be one of the most prevalent parasitic infestations globally. It is estimated that around 209 million people are affected by this condition, and it is somewhat more common in developing countries [1, 3]. Humans become infected through the fecal–oral route by ingesting the parasite's eggs from contaminated food or water. This disease is often asymptomatic but can sometimes manifest classically as nocturnal anal pruritus, especially in children. Approximately 20% of children worldwide have *E. vermicularis* infections.

*E. vermicularis* typically resides in the ileum and cecum. Once the eggs of *E. vermicularis* are ingested, they develop into adult worms in the small intestine within 1–2 months. When confined to the ileocecal area, the infection typically does not cause any symptoms. During the night, female adult worms and their eggs migrate to the anal area and deposit thousands of eggs in the perianal region, which causes pruritus. Perianal pruritus is caused by eggs hatching near the anal area, leading to contamination of the fingers and ingestion of the eggs (autoinfection), which restarts the life cycle of the worm. Occasionally, the larvae migrate back to the rectum and the small intestine, beginning the life cycle again (retroinfection) [3, 9].

Perianal itching is the most frequent manifestation of *E. vermicularis* infection, often occurring at night due to inflammation caused by adult worms and eggs on the perianal skin. Abdominal discomfort, nausea, and vomiting are other presentations that usually indicate a high worm burden [3]. Typically, the pathogenicity of *E. vermicularis* is mild, ranging from asymptomatic cases to nocturnal anal pruritus [1]. Our patient did not report any clinical symptoms of *E. vermicularis* infections, such as nocturnal itching.

*E. vermicularis* infection can cause diseases, such as AA, chronic appendicitis, ruptured appendicitis, gangrenous appendicitis, and perforation, resulting in peritonitis [7]. Fabrius first reported the occurrence of *E. vermicularis* infection in cases of AA in 1634 [10]. The prevalence of *E. vermicularis* infections causing AA in countries with high, upper-middle, lower-middle, and low-income levels was 3% (95% confidence interval [CI]: 2–4), 4% (95% CI: 1%–10%), 8% (95% CI: 1%–21%), and 1% (95% CI: 1–3), respectively. In Iran, the prevalence of AA caused by *E. vermicularis* is 1.5%–3.5%. Additionally, the prevalence of *E. vermicularis* infections causing AA in females (4.9%) was significantly higher than in males (2.7%) [11].

The histological findings commonly observed in resected appendiceal specimens vary from normal to inflammatory patterns, including lymphoid hyperplasia, eosinophilic infiltration, or neutrophilic infiltration. Macroscopically, helminths may be seen in the lumen of the appendix [1]. In our patient, despite the observation of worms in the lumen of the appendix, the microscopic appearance was normal.

There are two hypotheses regarding the possible mechanism of AA in individuals with parasitic infections. The first hypothesis suggests that the clinical signs of AA are caused by obstruction of the duct by adult parasites rather than true inflammation of the appendiceal wall [1]. The second hypothesis proposes that irregular migration of eggs and larvae can lead to the formation of granulomas in different parts of the body, such as the appendix, kidney [12], peritoneal cavity [13], male urinary tract [14], and female genital tract [15].

Although the gold standard treatment for AA is an appendectomy, this procedure merely addresses a complication while the root cause remains. Therefore, drug treatment should be administered to eradicate *E. vermicularis* [7]. The preferred drugs for treating *E. vermicularis* are mebendazole, pyrantel pamoate, and albendazole. Mebendazole is taken as a single 100 mg dose, with a repeat dose in 2 weeks. Pyrantel pamoate is given at a dose of 11 mg/kg, up to a maximum of 1 g, with doses administered 2 weeks apart. Albendazole is taken as a single 400 mg dose, with a repeat dose given in 2 weeks [16]. Treatment for *E. vermicularis* infection should be prescribed for both the patient and his/her family members. Additionally, patients must be educated on the importance of maintaining good hygiene practices and washing their hands regularly to prevent the spread of infection and reinfection [16].

## 4. Conclusion

In summary, histological patterns of AA associated with *E. vermicularis* are infrequent findings; however, *E. vermicularis* should be considered one of the differential diagnoses for the commonly presented condition of AA.

## Data Availability

Access to data is permitted with the author's permission.

## Nomenclature

AA: Acute appendicitis.
